# Museum of failed HIV research

**DOI:** 10.1080/13648470.2015.1079302

**Published:** 2015-12-03

**Authors:** Patricia Kingori, Salla Sariola

**Affiliations:** ^a^ The Ethox Centre, Nuffield Centre for Population Health, University of Oxford; ^b^ University of Turku, Finland

If a museum existed that contained a collection of failed artefacts related to HIV research what would that look like? How would failure be defined such that items could be representative of it and what would these definitions tell us about HIV research? These were some of the lines of enquiry which prompted the exploration of the *Museum of Failed HIV Research* as a conceptual space for scholars to examine these failure. This special issue is a collection of papers that have interrogated these questions and they present on display some of the possible ways of considering failure in HIV research.

The *Museum of Failed HIV Research* began its life as a panel at the Second International HIV Social Sciences and Humanities Conference in Paris, France, in July 2013. HIV has brought together various disciplines, e.g. Epidemiology, Global Health and clinical trials; qualitative health research; and Sociology, Science and Technology Studies, and Anthropology, and the conference reflected the range of languages, interests, styles and conventions of the multiple efforts targeting HIV/AIDS. The editors of this special issue invited scholars to submit papers that discussed failure. This forum framed the discussion of failure, and this special issue grew out of that panel in important ways. For instance, all these papers discuss contemporary examples of HIV research, which reflected the focus of conference. Additionally, and unlike the discussion of failure in biomedical fora, these papers employ a social scientific methodology, mainly ethnographic techniques, which is reflective of the host journal of this collection, *Anthropology* *&* *Medicine.*

The papers in this special issue examine a range of issues including community engagement, the role of volunteer labour in data production, evidence-based medicine, methodological concerns about the use of the case as the unit of analysis, developing caring relationships with research participants during the conduct of research and the role of ethics as a ‘fail-safe’ device in the conduct of HIV research and in regulating and promoting good practice. Unlike the exploration of historic discredited practices, these papers address ideas of contemporary importance. They all represent different areas of HIV research and practice which are currently deemed to be credible (Bloor 1976; Pels 2003). This has made the accounts stimulating as they have engaged in some of the ambiguities involved in ideas of failure and success. The contemporary nature of their subject-matter also presents opportunities to inform policy and practices in real-time. Furthermore, and unlike the study of controversies, none of the authors aimed to specifically examine failure in their research. However, through their work in HIV the concept of failure emerged in a variety of forms. As a consequence, all these papers employ the notion of failure as an analytical tool to open the possibility of considering HIV research and practice in alternative ways.

The papers in *Museum of Failed HIV Research* can be curated to address failure in multiple, overlapping ways. So rather than taking a position on whether these papers describe ideas, practices and processes that have objectively failed, we as editors see that the value of these papers is in what they discuss as failure and the arguments used to support these positions. Seeing these papers as a collection of artefacts in a museum, has allowed us as editors to put ourselves in the position of curators and to consider what types of narratives represent these accounts and for which audiences.

The papers could be curated according to how they define failure – in absolute or positivist terms or as socially constructed. Defining failure in positivist terms, considering something to have *actually* failed, allows assessments of success or failure to be made and why those occurred (such as in papers by Le Marcis, and Montgomery in this issue). This approach emphasises the ways in which failure can impact lives and practices (Campbell 2003). The biomedical literature also takes a positivist relationship with failure, which is often defined as negative findings. The growth in this literature has mirrored increasing pressure to make not only success but also failure public by reporting negative findings (Gupta and Stopfer 2011). This biomedical approach defines failure in very limited terms. In contrast, the papers in this collection have adopted a broader definition of failure to include systemic concerns, local social dynamics, inequalities and structural violence in keeping with the social science literature (e.g. Pigg 2013; Sariola 2009; Sobo 2009). For instance, in Sambakunsi et al.’s paper, examining the termination of employment of volunteer community counsellors in Malawi sees failure as being constructed.They present the working conditions of the unpaid volunteers who are tasked with fulfilling institutional self-testing targets in their community. They argue that volunteers' working conditions and the unrealistic nature of the targets are such that the volunteers' failure can be regarded as being to varying degrees constructed by their institutions.

A second way of curating the museum could be to emphasise the narrative that seemingly successful practices of HIV research and interventions can detract from areas and processes that can be deemed to be failures. For instance, Allman problematizes the ideologies and process involved in community engagement by showing that when participation becomes the gold-standard in social sciences, it marginalises theoretical analyses and undermines the value of conceptual thinking. In Cornish's work, systematic reviews of social interventions fail to take context into consideration, and she argues that abstracting community mobilisation is difficult, if not impossible. Cornish shows that there are very few examples of engagement available for comparison that are not tokenistic and that in most cases notions of evidence and other variables remain incompatible. Moreover, as papers by Allman, Cornish and Montgomery suggest, ideas of what success means, how prevention is conceptualised, and on whose terms, are dominated by the ‘biomedical’. From these papers those researching HIV from a social science perspective are shown to be under pressure to adopt practices and analytical strategies of biomedicine: Montgomery shows that a ‘case’ is an individualised approach to thinking about transmission, Cornish suggests that systematic reviews common to biomedicine when applied to social interventions are not meaningful tools for comparison and Allman is critical of the ways in which community engagement in social research marginalises social theoretical analyses. In this way, these papers have brought social science under the analytical lens of failure giving reflexive opportunities to a number of the disciplines involved in HIV research. As Nguyen's commentary highlights, this reflection represents the development and increasing maturity of the field of HIV research.

While it is possible to argue that bioethics has played a role in the development of ethical HIV research, there are papers in this collection that draw attention to the ways in which ethics plays an increasingly important role in defining success and failure in medical research. Papers by Peterson et al., Le Marcis, and Kingori show how current ethical practices can take attention away from local and global politics, de-politicising Global Health research. In this way, ethics and research regulation moves the question of failure to a ‘second’ register – ethics is put to task to ensure that science and technology are socially robust.

A third way of curating the museum could highlight the idea of *positionality* and how this shapes what is deemed to have failed (see also Timmermans 2011). The papers of this special issue show that there are various types of cadre involved in HIV research, ranging from volunteers to medical researchers, ethicists to social scientists and participating communities. Taking a cue from the social construction of technology and *interpretive flexibility* (Pinch and Bijker 1984), the perception of failure is highly dependent on the audience of the museum. In fact, it would be more appropriate to discuss *failures* in the plural because what failure means in a particular intervention or study can vary depending on the vantage point of the observer. Le Marcis shows how for study participants a trial can be a failure even if scientifically the treatment proves to work and is a success. Inversely, both Kingori and Montgomery show how a trial can be deemed to be a scientific failure but produce other outcomes that are regarded as being successful by and to participants. A number of papers have sought to identify some of the weaker actors in HIV research who are being held responsible for failure. For instance, Petersen et al. show how ethics committees in Malawi declined the PreP trial on three occasions. The authors argue that to present this as a failure of the ability of Malawian scientists and regulators to appreciate the potential of PreP research or as a failure of an African state more generally would be misleading. Instead, it should be understood from their position and concerns about imperial research ventures in an era of global off-shoring of research.

The articles in this special issue, with the exception of Nguyen's reflections on the last 30 years of the AIDS industry, cover a fairly recent history of HIV/AIDS. As artefacts in the museum, these papers are situated in an era marked by increasing emphasis on evidence and randomised controlled trials in both medicine and policy making (e.g. Wahlberg and McGoey 2007; Will and Moreira 2012). In search of larger data-sets, industry-sponsored clinical trials have shifted to low income settings, as have done Global Health projects, funded by academic and philanthropic sponsors, that draw various international actors together in collaboration or competition (Biehl and Petryna 2013; Crane 2013). While contemporary HIV research has been marked by globalised connections Q2 and assemblages (Ong and Collier 2005), it has also been mandated by expectations for social robustness (Corsín Jiménez 2005; Strathern 2005). ‘Mode 2’ knowledge production (Nowotny, Scott, and Gibbons 2001) is characterised by research that is socially relevant, ethically sound and engages communities. As HIV research and prevention projects have required bilateral and interdisciplinary efforts, HIV has been a driver of these trends as well as being defined by them.

All the papers in this special issue illuminate blind spots, of different kinds and across all the disciplines, involved in HIV research and practice. In this way, they remind us of the value of having a forum and space where multiple perspectives can be interrogated and contentious issues explored in ways that allow those participating in these spaces to consider and form their own impression of what is being presented. These papers do not diminish the importance of failure but rather reflect the complexities often found in identifying and gaining consensus in any absolute sense on what failure is, who is responsible for it and, in turn, what amounts to success.
